# DNA topoisomerase II alpha promotes the metastatic characteristics of glioma cells by transcriptionally activating β-catenin

**DOI:** 10.1080/21655979.2021.2023985

**Published:** 2022-01-11

**Authors:** Yi Liu, Jun Ma, Jiu-Shan Song, Hai-Ying Zhou, Jing-Hui Li, Cheng Luo, Xin Geng, He-Xiang Zhao

**Affiliations:** a Department II of Neurosurgery, The First Affiliated Hospital of Kunming Medical University, Kunming, Yunnan, China; b Pediatric Department, The First Affiliated Hospital of Kunming Medical University, Kunming, Yunnan, China

**Keywords:** TOP2A, β-catenin, invasion, glioma, MMPs

## Abstract

DNA topoisomerase II alpha (TOP2A) reportedly plays a crucial role in several cancers, however, the precise regulatory role of TOP2A in metastatic characteristics of glioma is still poorly understood. Herein, we sought to elucidate the mechanisms by which TOP2A affects the metastatic phenotypes of glioma. We observed that a high level of TOP2A expression was dramatically linked with inferior survival in glioma patients while silencing of TOP2A impaired glioma cell proliferation and aggressiveness. TOP2A was found to directly interact with β-catenin and facilitated its translocation into the nucleus. Mechanistically, TOP2A effectively induced glioma cell growth and invasion in a β-catenin-dependent manner. Overall, we pinpoint TOP2A as a critical activator of the Wnt/β-catenin pathway in glioma, promoting cell growth, migration, and invasion.

## Introduction

Glioma is the most frequent and aggressive neoplasm in the brain [1]. Currently, the treatment options for glioma include surgical excision, followed by radiotherapy and chemotherapy with temozolomide [2]. The high rates of local invasion and metastasis are the main reasons for the poor clinical outcome of patients with glioma [3]. Therefore, it is imperative to exploit the precise molecular mechanism in the metastatic process to provide valuable prognostic indicators and potential therapeutic targets.

The metastatic glioma cell undergoes a multi-step cell-biological process that facilitates extracellular matrix adhesion, secretion of matrix metalloproteinases (MMPs) that degrade the matrix surrounding tumor cells, migration through the glioma microenvironment, and formation of metastatic lesions [4,5]. The expressions of MMPs, including MMP-2 and MMP-9, have been shown to coordinate calcium mobilization and induce glioma cell metastasis [6]. Dozens of investigations have explored the molecular regulatory mechanisms contributing to glioma invasion and metastasis.

Emerging studies have delineated that TOP2A is aberrantly expressed in various solid tumors, including cervical cancer, bladder urothelial carcinoma, and lung adenocarcinoma [7–9]. High expression of TOP2A is closely associated with an unfavorable prognosis in hepatocellular carcinoma [10]. The clinical and prognostic values of TOP2A in breast carcinoma, nasopharyngeal carcinoma, and pancreatic cancer have also been sufficiently validated [6,11,12]. Although TOP2A has been shown to play crucial roles in cancers, it is imperative to investigate the pro-metastasizing function of TOP2A in glioma.

In the current report, we aim to study the potential role of TOP2A in glioma and the underlying molecular mechanism. We corroborated that TOP2A is noticeably upregulated and high TOP2A expression was dramatically associated with inferior prognosis in glioma patients. Furthermore, TOP2A depletion attenuated the metastatic potential of glioma cells. Our results highlighted that ectopic TOP2A overexpression augmented the expression of β-catenin and promoted its translocation into the nucleus. In sum, we clarified the underlying role of β-catenin and the oncogenic function of TOP2A in glioma.

## Materials and methods

### Cell culture

Normal brain glial cells (HEB) and glioma cell lines (U251, U-87 MG, TJ905 and T98G) were bought from KeyGen Biotech Co., Ltd (Nanjing, China). All cells were cultured in Dulbecco’s Modified Eagle Medium (DMEM) supplemented with 10% fetal bovine serum (FBS) in a 5% CO_2_ atmosphere at 37℃. Aclarubicin hydrochloride and XAV939 were obtained from MedChemExpress (Shanghai, China).

### siRNA or plasmid transfection

The short hairpin RNA (shRNA) against TOP2A (sh-TOP2A) and small interfering RNA (siRNA) targeting β-catenin (si-β-catenin) were synthesized by RiboBio (Guangzhou, China). U-87 MG or T98G cells were transfected with sh-TOP2A or scrambled control (sh-Con) utilizing the Lipofectamine 2000 kit (Thermo Fisher Scientific). Human TOP2A overexpressed plasmid (TOP2A) and control vector were obtained from GeneChem (Shanghai, China). After 48 h, transfected cells were subjected to Western blot assay. Lentivirus with shRNA against TOP2A and negative shRNA were provided by GeneCopoeia (Guangzhou, China). T98G cells were transduced with lentiviral shRNA according to the protocol, and cells were selected in puromycin (2.5 mg/ml) to obtain a stable transduced cell line.

### Bioinformatics analysis

We analyzed The Cancer Genome Atlas (TCGA) data portal to compare the expression pattern of TOP2A in glioma samples and normal control using Gene Expression Profiling Interactive Analysis (GEPIA) tool (http://gepia.cancer-pku.cn/). Additionally, overall survival (OS) and disease-free survival (DFS) from patients with glioma were analyzed with the help of GEPIA [13].

### RNA extraction and quantitative real-time PCR (qRT-PCR)

Total RNA in U-87 MG or T98G cell was extracted by using a TRIzol kit (Thermo Fisher Scientific) and utilized to synthesize complementary DNA (cDNA) with a PrimeScript RT Reagent Kit (TaKaRa, Japan). qRT-PCR was implemented on an iCycler real-time PCR instrument (Bio-Rad) with a SYBR Green PCR Master Mix (TaKaRa). The PCR primers were listed as following: glyceraldehyde-3-phosphate dehydrogenase (GAPDH) (forward), 5′-GGAGCGAGATCCCTCCAAAAT-3′, GAPDH (reverse), 5′ GGCTGTTGTCATACTTCTCATGG-3′; TOP2A (forward), 5′-ACCATTGCAGCCTGTAAATGA-3′, TOP2A (reverse), 5′-GGGCGGAGCAAAATATGTTCC-3′; MMP-2 (forward), 5′-GTATTTGATGGCATCGCTCA-3′, MMP-2 (reverse), 5′-CATTCCCTGCAAAGAACACA-3′; MMP-9 (forward), 5′-GATGCGTGGAGAGTCGAAAT-3′, MMP-9 (reverse), 5′-CACCAAACTGGATGACGATG-3′; β-catenin (forward), 5′-AAAGCGGCTGTTAGTCACTGG-3′, β-catenin (reverse), 5′-CGAGTCATTGCATACTGTCCAT-3′. The relative level was determined using the 2^−ΔΔCt^ method and normalized to the GAPDH level.

### Immunoblotting

Cell lysates in U-87 MG or T98G cells were extracted, and 10% sodium dodecyl sulfate-polyacrylamide gel electrophoresis (SDS-PAGE) was used to separate total-protein. The nuclear and cytoplasmic extract was prepared using a NE-PER™ Nuclear and Cytoplasmic Extraction Reagent (Thermo Fisher Scientific). After being transferred onto polyvinylidene fluoride (PVDF) membranes, bands were detected after incubation with a primary antibody overnight at 4°C followed by horseradish peroxidase (HRP)-conjugated anti-rabbit secondary antibody for 2 h. All blots were visualized using an enhanced chemiluminescence (ECL) kit (Millipore). Anti-TOP2A, anti-β-catenin, anti-GAPDH, and secondary antibodies were purchased from Santa Cruz Biotechnology (Santa Cruz, CA, USA).

### Luciferase reporter assay

The human β-catenin promoter region was cloned into the NheI/BglII sites of pGL3- luciferase reporter plasmid (Promega). U-87 MG or T98G cells (3 × 10^4^ cells/well) were plated into 96-well plates. pRL-TK Renilla plasmid (Promega) and pGL3 plasmid were co-transfected into cells with a Lipofectamine 3000 kit. 48 h post-transfection, luciferase activities were detected using a dual-luciferase assay kit (Promega).

### Co-immunoprecipitation (Co-IP)

Cell lysates were prepared from U-87 MG or T98G cells using radioimmunoprecipitation (RIPA) lysis and incubated with anti-TOP2A or IgG overnight at 4°C. Subsequently, the mixture was incubated with protein G beads (Abcam) at room temperature for 3 h. After centrifugation, the precipitated complex was subjected to Western blotting [14].

### Cell counting kit-8 (CCK-8)

Cells (2 × 10^3^) were seeded on 96-well plates. After cells incubation according to indicated times, 10 μL of CCK-8 solution (Dojindo, Kumamoto, Japan) was added to the plates. After 2 hours in an incubator, optical density (OD) value at 450 nm was measured using a microplate reader.

### Clone-formation analysis

Cells (1 × 10^3^) were seeded onto 6-well plates to allow colony formation. After 14 days, colonies were dyed with crystal violet. The number of colonies was then quantified under an inverted microscope.

### Migration and invasion assay

24-well Transwell chambers (8-μm pore size) coated or not-coated with Matrigel (BD Biosciences, USA) were used to detect cell migration or invasion, respectively. 100 μL of cell suspension (2 × 10^3^) was added into the upper chamber, and the lower chamber was filled with 500 μL of medium containing 10% FBS. After 24 h, cells were fixed with 4% paraformaldehyde and dyed with 1% crystal violet. The number of cells across the filter was counted using an inverted microscope [15].

### MMP enzyme-linked immunosorbent assay (ELISA)

Specific MMP enzymatic activities were determined as previously described in conditioned media using a MMPs ELISA Kit for MMP-2 and −9 (Thermo Fisher Scientific) [16]. The whole procedure to measure the total activity of MMP-2/9 was performed according to the manufacturer’s instructions.

### Xenograft model

100 μL of sh-NC or sh-TOP2A stably transduced T98G cells (2 × 10^6^) were subcutaneously inoculated into Balb/c nude (6 mice in each group). The width and length of tumor size were measured every week with a caliper. Tumor volume = length×width^2^/2. After 28 days, mice were sacrificed, and excised tumors were subjected to immunohistochemical (IHC) staining using MMP-2/9 antibody. This study was approved by the Institutional Animal Care and Use Committee of The First Affiliated Hospital of Kunming Medical University.

### Statistical analysis

Statistical analysis was conducted using GraphPad Prism 8.0. All experiments were performed at least three times, and the mean values ± standard deviation (Mean ± SD) were determined from triplicate samples for each treatment group. The difference was analyzed using a one-way analysis of variance (ANOVA) or t-test. A P-value of less than 0.05 was deemed significant.

## Results

This study revealed that TOP2A expression is aberrantly highly expressed in glioma, and abnormally high TOP2A expression could serve as a prognostic indicator. TOP2A depletion attenuated the metastatic potential of glioma cells *via* promoting the translocation of β-catenin into the nucleus.

### Silencing of TOP2A restrains glioma cell growth

**Figure 1.**
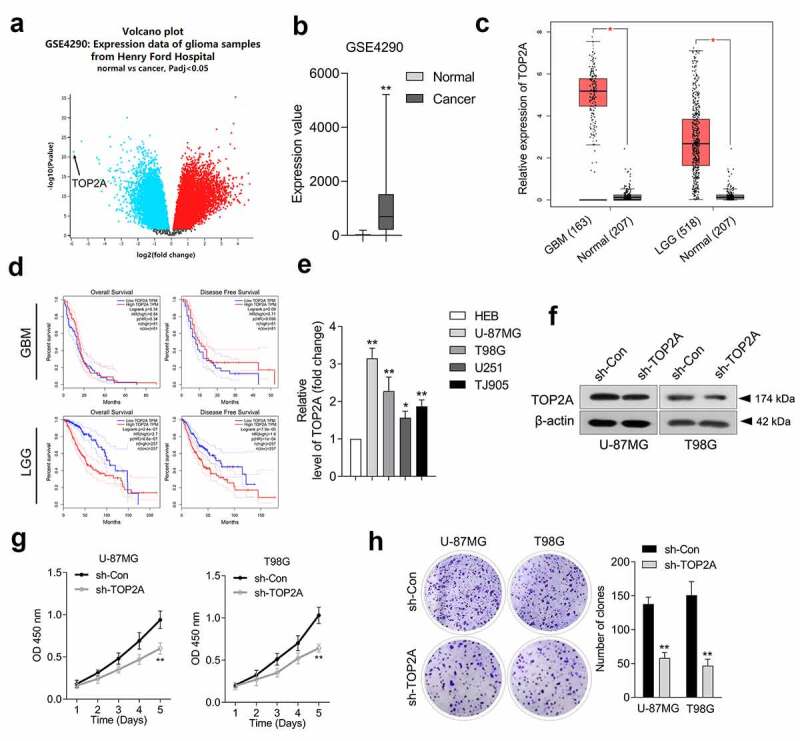
TOP2A is overexpressed in glioma tissues and cells. (a) We analyzed differentially expressed genes in GSE4290 microarray dataset. Volcano plot of significant differentially expressed genes with |logFC|> 2 and *P* < 0.05. (b) The abnormal TOP2A expression was presented as box-plot diagram. (c) TOP2A expression was elevated in glioblastoma (GBM) and low-grade glioma (LGG) compared with normal using TCGA database (https://tcga-data.nci.nih.gov/tcga/). (d) Upper panel: Kaplan-Meier analysis of overall survival (OS) and disease-free survival (DFS) in GBM patients with low TOP2A expression (81) and high TOP2A expression (n = 81). Lower panel: Kaplan-Meier analysis of OS and DFS in LGG patients with low TOP2A expression (257) and high TOP2A expression (n = 257). (e) Relative mRNA levels of TOP2A in normal brain glial cell line, HEB and glioma cell lines. (f) TOP2A expressions in U-87 MG and T98G cells transfected with sh-TOP2A were detected using Western blot. (g) The proliferation of sh-TOP2A transfected U-87 MG and T98G cells were determined using CCK-8 assay. (h) Colony formation analysis of glioma cells growth. Data were determined from triplicate experiments (Mean ± SD). **P < 0.01 compared with sh-Con.

According to Gene Expression Omnibus (GEO) profiling data (GSE4290), TOP2A exhibits a higher level in glioma than in normal tissues (Figure 1(a-b)). TOP2A mRNA expression level data was extracted from the TCGA dataset using the GEPIA platform. Similarly, glioma tissues, including glioblastoma (GBM) and low-grade glioma (LGG), exhibited higher expressions of TOP2A (Figure 1(c)). Overall survival of TOP2A expression in glioma patients was identified from the TCGA database. As indicated in Figure 1(d), the elevated expression of TOP2A was associated with inferior OS and DFS in LGG patients. However, there was no correlation between the altered expression of TOP2A and OS as well as DFS in GBM patients. Finally, TOP2A was significantly upregulated in glioma cell lines compared to HEB cells (Figure 1(e)). U-87 MG and T98G cells exhibited higher protein expression of TOP2A. Thus, a loss-of-function assay was implemented using U-87 MG and T98G to elaborate the biological significance of TOP2A. TOP2A shRNA (sh-TOP2A) stably silenced TOP2A expression in U-87 MG and T98G cells (Figure 1(f)). Results from CCK-8 and clonogenic assays indicate that TOP2A deletion profoundly reduces cell proliferation (Figure 1(g)) and clonal formation (Figure 1(h)) ability in U-87 MG and T98G.

### Attenuation of TOP2A expression inhibits glioma cell migration and invasion

**Figure 2.**
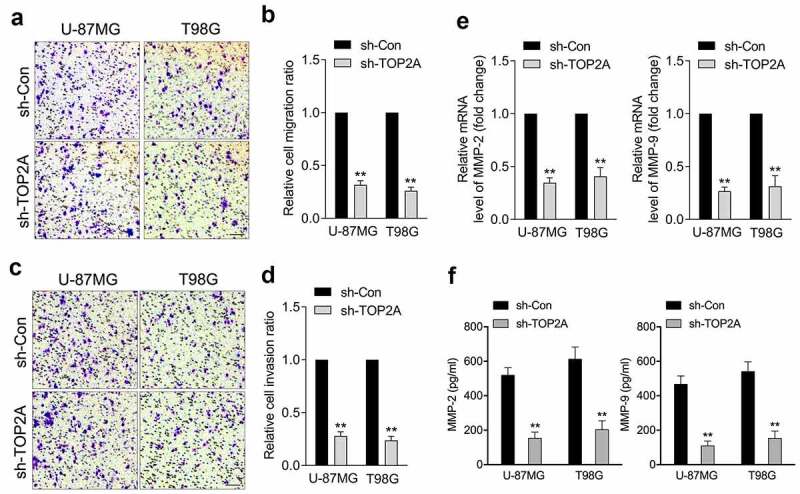
Silencing of TOP2A inhibits glioma cell migration and invasion in vitro. (a-b) Representative and quantification results of transwell migration assay in U-87 MG and T98G cells transfected with sh-TOP2A. (c-d) Representative and quantification results of transwell invaison assay in U-87 MG and T98G cells transfected with sh-TOP2A. (e) The mRNA levels of MMP-2/9 were detected using qRT-PCR. (f) Determination of MMP-2 and MMP-9 enzymatic activity by ELISA assay. Data were determined from triplicate experiments (Mean ± SD). **P < 0.01 compared with sh-Con.

A typical feature of glioma is diffuse tumor invasion, and we sought to determine the biological significance of TOP2A in glioma migration/invasion. As illustrated in Figure 2(a-d), a decrease in TOP2A expression was accompanied by a decrease in U-87 MG and T98G cell migration and invasion. Aberrant MMP-2 and MMP-9 activities have been implicated in the deterioration of glioma. TOP2A knockdown dramatically decreased the mRNA levels and activities of MMP-2 and MMP-9 in U-87 MG and T98G cells, as documented by qRT-PCR (Figure 2(e)) and ELISA assay (Figure 2(f)), respectively.

### TOP2A physically interacts with β-catenin

**Figure 3.**
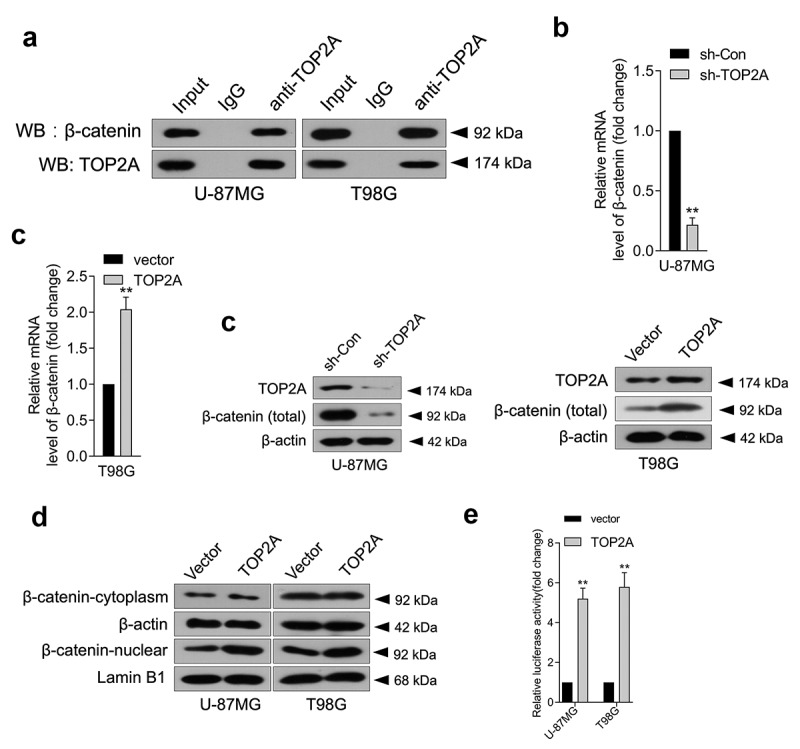
TOP2A facilitates β-catenin entry into the nucleus, and TOP2A promotes the transcription of β-catenin. (a) Co-immunoprecipitation assay showed that TOP2A could bind to β-catenin. (b-c) The mRNA levels and protein expressions of β-catenin were detected using qRT-PCR or Western blot, respectively. (d) The change in distribution of β-catenin in U-87 MG and T98G cells transfected by TOP2A overexpression plasmid or vector. (e) Overexpression of TOP2A stimulated the luciferase reporter activity of β-catenin promoter reporter. **P < 0.01 compared with vector.

In pancreatic cancer, TOP2A was found to promote malignancy *via* activating β-catenin [6]. Aberrantly activated Wnt/β-catenin signaling has been detected in various malignancies, including glioma [17,18]. To clarify the mechanism by which TOP2A influences glioma cell growth, migration, and invasion, a Co-IP assay was carried out to ascertain the interaction between β-catenin and TOP2A in glioma. As displayed in Figure 3(a), TOP2A co-immunoprecipitated with endogenous β-catenin. Furthermore, the mRNA level (Figure 3(b)) and protein expression (Figure 3(c)) of β-catenin decreased upon TOP2A knockdown and increased when TOP2A was introduced into U-87 MG and T98G cells. Particularly, β-catenin levels were enriched in the nucleus when TOP2A expression was raised, which indicated that TOP2A increased β-catenin entry into the nucleus (Figure 3(d)). To ascertain whether TOP2A can directly bind to the promoter region of β-catenin and thus regulate β-catenin expression, a firefly luciferase reporter gene vector containing β-catenin promoter fragment was constructed. In U-87 MG and T98G cells, TOP2A overexpression significantly activated the luciferase activity of full-length β-catenin promoter (Figure 3(e)). Taken together, these results demonstrated that TOP2A could mainly regulate β-catenin promoter activity by binding to the β-catenin promoter region.

### β-catenin mediates the tumor-promoting effect of TOP2A

**Figure 4.**
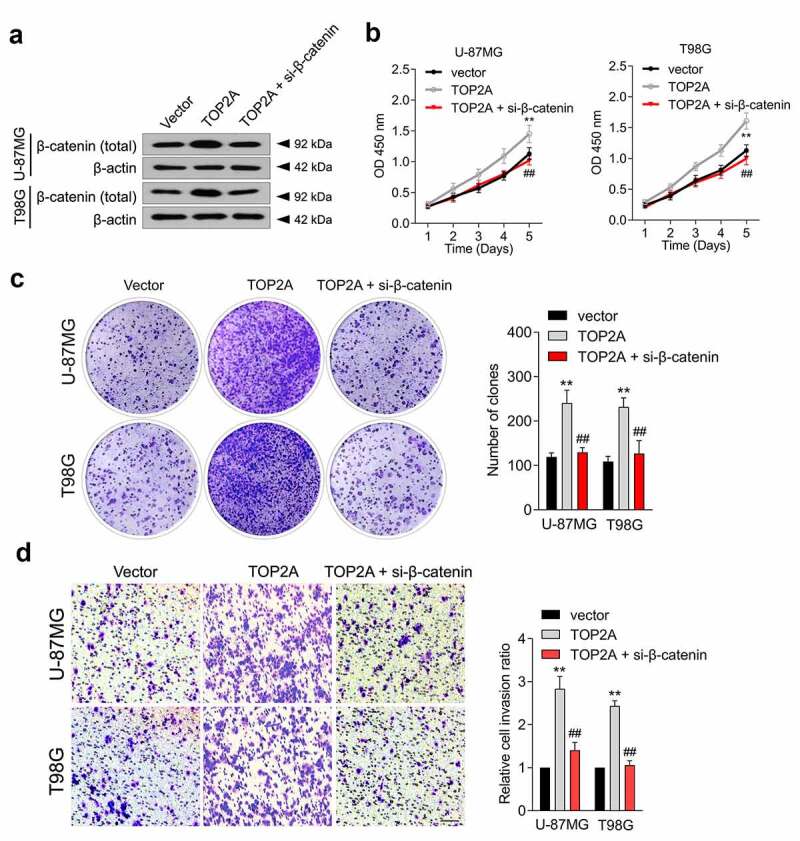
β-catenin mediates the function of TOP2A to promote the proliferation and invasion of glioma cell. (a) U-87 MG and T98G cells were transfected with TOP2A or cotransfected with TOP2A and si-β-catenin. The expression of β-catenin was measured using immunoblotting. (b) The growth curves of indicated cells were detected by CCK-8 assay. (c) The colonizing abilities were detected by colony formation assays when β-catenin was knocked down in cells transduced with TOP2A. (d) The invasion abilities were assessed by transwell assays when β-catenin was knocked down in cells transduced with TOP2A plasmid. Data were determined from triplicate experiments (Mean ± SD). **P < 0.01 compared with vector, ^##^*P* < 0.01 compared with TOP2A.

To validate whether β-catenin is implicated in the cancer-promoting effect of TOP2A, si-β-catenin was introduced into TOP2A-overexpressed U-87 MG and T98G cells (Figure 4(a)). Silencing β-catenin decreased TOP2A-induced cancer cell proliferation (Figure 4(b)), colony formation (Figure 4(c)), and invasion (Figure 4(d)). Moreover, the effect of TOP2A inhibitor (aclarubicin hydrochloride) on cell migration and invasion, alone and in combination with the β-catenin inhibitor, XAV939, in T98G cells were evaluated [19–21]. As shown in Supplementary Figure 1, 0.1 μM aclarubicin hydrochloride and 1 μM XAV939 resulted in marked suppression of the migratory and invasive abilities of T98G cells. The combination treatment with aclarubicin hydrochloride and XAV939 showed more inhibited cell migration and invasion compared to each treatment alone. These observations indicated that TOP2A could affect glioma cell growth and metastatic properties in a β-catenin-dependent manner.

### Knockdown of TOP2A impairs the oncogenicity of glioma cells in vivo

**Figure 5.**
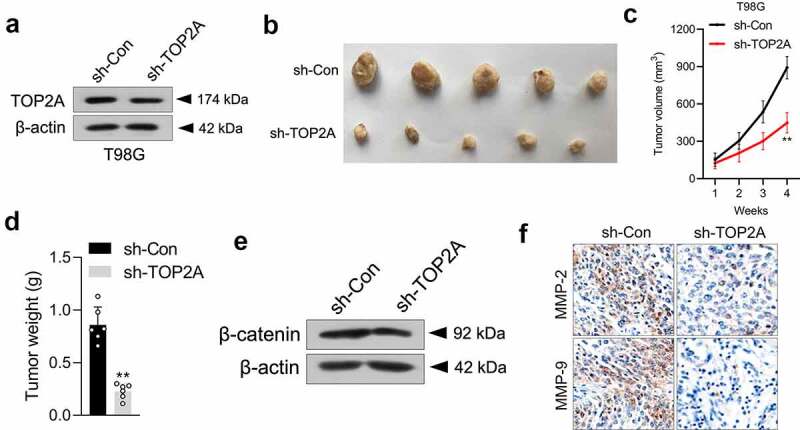
Silencing of TOP2A suppresses T98G cell growth in vivo. (a) Western blot analysis of TOP2A expression in sh-Con or sh-TOP2A stably transfected T98G cells. (b) Photographs of the harvested xenograft tumors at 28 days post-implantation. (c-d) The volumes and weights of tumors from tumor-bearing nude mice injected with sh-Con or sh-TOP2A transfected T98G cells. (e) The levels of β-catenin expression in tumor tissues formed from sh-Con or sh-TOP2A transfected T98G cells were determined by immunoblotting. (f) IHC staining of MMP-2/9 in tumor tissues. Scale bar: 100 μm. **P < 0.01 compared with sh-Con.

To illuminate the biological significance of TOP2A in an animal experiment, a stably transduced sh-TOP2A T98G cell line was constructed (Figure 5(a)) and then subcutaneously inoculated into nude mice. As expected, the sh-TOP2A group exhibited smaller tumor volumes and weights (Figure 5(b-d)). Then, the expressions of β-catenin in xenograft tissues were checked *via* Western blot, which showed that β-catenin expression was markedly suppressed in sh-TOP2A transfected T98G cells formed xenograft tumors (Figure 5(e)). Finally, the results from IHC staining further confirmed the alterations in MMP-2/9 expressions caused by sh-TOP2A (Figure 5(f)).

## Discussion

The pathogenesis of glioma is a complicated process that involves abnormal expressions of anti-oncogenes or oncogenes [22]. An extensive body of investigations identified innovative cancer-related genes *via* sequencing technology [23]. In our study, GEO datasets in combination with TCGA data suggested that TOP2A expression was profoundly elevated in glioma. Additionally, TOP2A was found to be a prognostic factor for patients with glioma. These results hint at a potential involvement of TOP2A in glioma development.

Metastasis is the main trigger for poor prognosis in glioma [24,25]. TOP2A, as one of the two members of the topoisomerase II (TOP2) family, is expressed only in proliferating cells and is responsible for enzymatic uncoupling during the replication of DNA strands [26]. TOP2A catalyzes double-stranded DNA breaking and induces gene transcription during mitosis [27]. The correlation between TOP2A and cancer development has been explored in several cancers [7,28,29]. With the assistance of bioinformatic analysis, Zhou et al. corroborated that over-expression of TOP2A is linked with a worse prognosis in glioma patients [30]. TOP2A promotes cervical cancer cell migration, invasion, and epithelial-mesenchymal transition (EMT) *via* activating the phosphatidylinositol-3-kinase (PI3K)/V-akt murine thymoma viral oncogene homolog (AKT) signal [7]. It has been previously verified that miR-144-3p could induce glioma cell apoptosis and suppress cell migration by modulating TOP2A [31]. However, the pathological role of TOP2A is not well defined for glioma. In our study, inhibition of TOP2A reduced the proliferation, migration, and invasive capacities of glioma cells. Strong correlations have been reported between elevated MMPs levels and tumor cell invasiveness in human glioma [32]. Silencing TOP2A drastically led to the downregulation of MMP-2/9 mRNA levels and activities. In short, these findings showed that TOP2A has a pro-oncogenic role in glioma

Aberrant Wnt/β-catenin signaling activation boosts the invasion and metastasis of various malignancies, including glioma [33,34]. In this current study, the results of Co-IP assays combined with the dual-luciferase reporter system showed that TOP2A could directly bind to β-catenin and reinforce the transcriptional activity of the β-catenin promoter. Furthermore, the mRNA level and protein expression of β-catenin were raised by TOP2A overexpression and were decreased when TOP2A was deleted in glioma cells. Subsequently, TOP2A could facilitate the nuclear translocation of β-catenin and reinforce the luciferase activity of β-catenin promoter, indicating that TOP2A could function as an activator of Wnt/β-catenin signaling.

Herein, after β-catenin deletion in the TOP2A-overexpressing glioma cell, the growth and aggressive traits were partly retained. These outcomes demonstrated that the pro-metastatic ability of TOP2A in glioma cells was dependent on β-catenin, which in turn mediated the cancer-promoting role of TOP2A. However, our study has several limitations: a further study on how TOP2A binds to the β-catenin promoter to transcriptionally activate gene expression is still required. Future experiments, such as promoter truncation assay and chromatin immunoprecipitation (ChIP)-quantitative PCR require further analysis of specific transcriptional binding sites of TOP2A in the β-catenin promoter. In addition, the mechanisms underlying TOP2A overexpression in glioma samples also remain to be elucidated.

## Conclusion

In sum, TOP2A physically binds with β-catenin and facilitates β-catenin entry into the nucleus of glioma cells. TOP2A effectively intensifies glioma cell metastatic phenotypes in a β-catenin-dependent manner. This study presents the first evidence that TOP2A has an important role in promoting glioma cells, reinforcing the view that TOP2A is a critical factor in glioma development.

## Supplementary Material

Supplemental Material

## Data Availability

The datasets used or/and analyzed during the current study are available from the corresponding author on reasonable request.
