# Supplementary material for: DNA topoisomerase II alpha promotes the metastatic characteristics of glioma cells by transcriptionally activating β-catenin
Source: Bioengineered. 2022 Jan 11;13(2):2207–16. doi: 10.1080/21655979.2021.2023985 (PMC8974225; doi:10.1080/21655979.2021.2023985)
Supplement: Supplemental Material [file KBIE_A_2023985_SM4110.zip › supplementary/supplementary materials.docx]

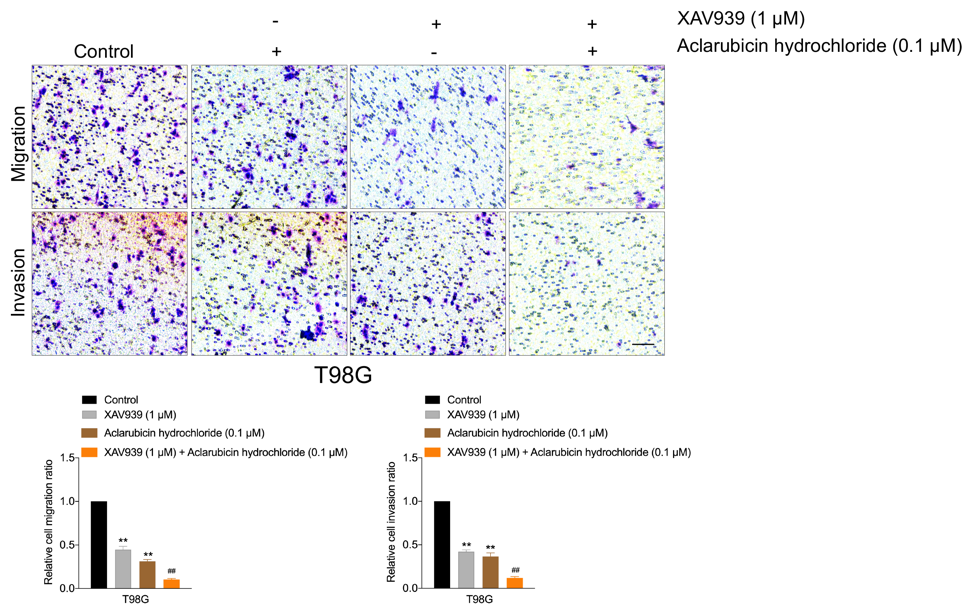


Supplementary Figure 1. Combination treatment with aclarubicin hydrochloride (0.1 μM) and XAV939 (1 μM) inhibited the migration and invasion of T98G cells. Migration/invasion assay of T98G cells treated with aclarubicin hydrochloride and/or XAV939 for 24 h. Migrated or invaded cells were stained with crystal violet and photographed. Bar graph shows quantitative data. ^**^*P*<0.01 compared with control, ^##^*P*<0.01 compared with XAV939 (1 μM) group.
